# Paclitaxel- or sirolimus-coated balloons used for ArterioVEnous fistulas-2 (PAVE-2): study protocol for a randomised controlled trial to determine the efficacy of paclitaxel- or sirolimus-coated balloons in arteriovenous fistulas used for haemodialysis

**DOI:** 10.1186/s13063-024-08502-1

**Published:** 2024-10-31

**Authors:** Narayan Karunanithy, Sam Norton, Francis Calder, Neelanjan Das, Niamh Dooley, Lusine Hakobyan, Robert Jones, Soundrie Padayache, Chloe Spriggs, Kate Steiner, Rebecca Suckling, Michael G. Robson

**Affiliations:** 1 Guy’s and St Thomas’ NHS Foundation Trust, London, SE1 9RT UK; 2 King’s College London, London, SE1 9RT UK; 3 East Kent University Hospitals NHS Trust, Canterbury, CT1 3NG UK; 4 University Hospitals Birmingham NHS Foundation Trust, Birmingham, B15 2GW UK; 5 East and North Hertfordshire NHS Trust, Stevenage, SG1 4AB UK; 6 Epsom and St, Helier University Hospitals NHS Trust, Carshalton, SM5 1AA UK

**Keywords:** Haemodialysis, Arteriovenous fistula, Angioplasty, Fistuloplasty, Paclitaxel, Sirolimus

## Abstract

**Background:**

In view of the conflicting results from previous studies, the benefit of paclitaxel-coated balloons for arteriovenous fistulas is uncertain and equipoise remains. Although an industry-led trial testing the efficacy of sirolimus-coated balloons in AVFs is in progress, the benefit of sirolimus-coated balloons for arteriovenous fistulas is currently unknown. The purpose of this trial is to compare the efficacy of additional paclitaxel-coated or sirolimus-coated balloons on outcomes after a plain balloon fistuloplasty to preserve the patency of arteriovenous fistulae used for haemodialysis.

**Methods:**

The study design is a multicentre randomised controlled trial. Following a successful plain balloon fistuloplasty, participants will be randomised to further treatment with a paclitaxel-coated balloon, a sirolimus-coated balloon, or an uncoated control balloon. We will recruit 642 patients, each with one or two treatment segments, over a 3-year period. Patients will remain in the trial and be followed up for 1 year. The primary endpoint is time to loss of treatment segment primary patency. Cox-proportional hazards models will be used to estimate hazard ratios for the time to loss of treatment segment primary patency for each treatment group relative to the control group. Analysis of the primary endpoint will be based on treatment segments rather than participants and a shared frailty will be estimated to account for the clustering of treatment segments within patients. Secondary endpoints are time to loss of primary patency at any treatment segment; time to end of access circuit primary patency; time to AVF abandonment; number of radiological or surgical interventions; adverse events; intima-media thickness and degree of stenosis at 3 months on ultrasound; and patient quality of life assessed by EQ-5D-5L and VASQoL.

**Discussion:**

The three-armed design in this proposal will provide an answer on the efficacy of both paclitaxel- and sirolimus-coated balloons in the same trial. This trial is likely to provide a clear answer regarding the efficacy of drug-coated balloons for arteriovenous fistulas.

**Trial registration:**

ISRCTN ISRCTN40182296. Registered on 4 August 2023.

## Background

### The clinical need being addressed

Vascular access has been described as the Achilles heel of haemodialysis. Reliable access is essential to provide treatment, but arteriovenous fistulas (AVFs) and arteriovenous grafts (AVGs) frequently develop stenoses which affect their function. This causes ineffective dialysis treatment, hospital admissions and disruption to the lives of patients. The Standardised Outcomes in Nephrology-Hemodialysis (SONG-HD) initiative was an international consensus process involving > 1300 patients, caregivers and health professionals from more than 70 countries [1]. Vascular access was identified as the most important issue. The James Lind Alliance Priority Setting Partnership (JLA PSP) has published the top 10 priorities in vascular access research [2]. These were agreed upon in July 2021 at a final workshop for patients and health care professionals. The number one priority was “What can be done to make fistulas or grafts last as long as possible?” The PAVE-2 trial directly addresses this question, with an intervention with the potential to prolong the patency of AVFs.

In the UK, 37.8% of the 68,111 patients on renal replacement therapy receive haemodialysis [3]. Based on the number of fistuloplasties per patient per year at Guy’s and St Thomas’ and other centres, we estimate that over 5000 fistuloplasties per year are performed in the UK. AVFs are the best form of vascular access for haemodialysis but they have a limited survival. A fistuloplasty is an effective treatment for stenosis. It is required in 50% of patients before the fistula is used [4] and the majority of patients receiving haemodialysis will need a fistuloplasty at some point while a fistula is in use. Post-intervention primary patency rates are around 60–70% at 6 months and 40–50% at 1 year [5–11], and so repeat intervention is commonly required. Therefore, there is a need to identify treatments that prolong the time to reintervention following a fistuloplasty. Currently, drug-coated angioplasty balloons are rarely used in AVFs in routine clinical practice.

Hospital admission rates due to both infection and access problems are lowest in patients with an AVF compared to a central venous catheter (CVC) or arteriovenous graft (AVG) [12]. Haemodialysis patients are at a greatly increased risk of infection with invasive *Staphylococcus aureus*—methicillin-resistant (MRSA) or methicillin-sensitive (MSSA)—and this is largely related to the use of CVC. The risk of invasive infection is increased 100-fold over the general population in haemodialysis patients, with 85% having CVCs, and 90% requiring hospitalisation with a 17% mortality [13]. A UK study showed that the risk of MRSA bacteraemia was approximately 4.5 times higher with a CVC than an AVF and the risk of MSSA infection was around 3.3 times higher [14]. In addition to infections, CVCs cause central venous stenosis. A retrospective study of over 100 patients showed that the cumulative risk at 1 year of CVC-associated bacteraemia or central venous stenosis was 9% and 2%, respectively [15].

Therefore, interventions that prolong the survival of AVFs would reduce the use of CVCs with associated complications, in addition to decreasing the need for further radiological or surgical procedures. There would also be fewer hospital admissions, an improved quality of life for patients and significant cost savings for the NHS.

### Existing research

Paclitaxel-coated balloons allow local delivery of paclitaxel to the site of stenosis. Paclitaxel is a drug which inhibits the cellular proliferation leading to restenosis following fistuloplasty. Since 2015 there have been many studies published assessing their effectiveness in preserving patency following angioplasty of an AVF. A systematic review, published in February 2022, concluded that there was no evidence of benefit [16]. Many studies included in the above metanalysis had a small sample size and there was considerable heterogeneity. There have been three multi-centre randomised controlled trials (RCTs) with more than 200 participants. These three studies were all high quality, and each had significantly more participants than other studies. Therefore, it is useful to consider the findings of these three studies separately. The first was from Trerotola, sponsored by industry (285 participants) and had a primary endpoint of target lesion primary patency (TLPP) at 6 months. There was no significant difference between groups treated with a paclitaxel-coated balloon compared with a control group receiving treatment with a non-coated balloon [17]. A second industry-sponsored study by Lookstein (330 participants), using the same binary primary endpoint but a different paclitaxel-coated balloon, did find a difference between groups [18]. However, the investigator-led, UK-wide, NIHR-EME funded Paclitaxel-assisted balloon Angioplasty of Venous stenosis in hEmodialysis access (PAVE) trial (212 participants) failed to show an effect on time to end of TLPP after treatment with a Lutonix paclitaxel-coated balloon compared with a non-coated balloon [19]. The reason that the PAVE trial and the study by Trerotola did not show a benefit whereas the study by Lookstein did is not entirely clear. A possible explanation is that the two negative trials used the Lutonix balloon, whereas the Lookstein study used the IN.PACT balloon.

Sirolimus is another anti-proliferative drug that may be of benefit in this setting. Several lines of evidence suggest that sirolimus may prevent the neointimal hyperplasia that causes arteriovenous fistula restenosis. Firstly, sirolimus is an anti-proliferative drug and has been shown to inhibit both proliferation and migration in vascular smooth muscle cells in vitro [20]. Smooth muscle cell proliferation and migration cause neointimal hyperplasia and stenosis in AVFs [21]. Secondly, sirolimus has been shown to prevent venous neointimal hyperplasia in murine vein grafts in vivo [22]. More recently, sirolimus was shown to inhibit neointimal hyperplasia and improve patency in a murine AVF model [23].

Sirolimus-coated balloons have now been developed and are therefore an alternative to paclitaxel-coated balloons. The 6- and 12-month results from a single-arm study (MATILDA) study showed promising results. The target lesion primary patency rates at 3 and 6 months were 46/47 (97.9%) and 29/35 (82.9%) respectively [24]. At 12 months, TLPP was 22/38 (58%) [25]. Results from 6- and 12-month results from a second single-arm study (ISABELLA) using a different device have been published [26–28]. Target lesion primary patency rates at 3, 6 and 12 months were 39/41 (95.1%), 28/39 (71.8%) and 16/36 (44.4%), respectively. The industry-sponsored RCT (IMPRESSION), recruiting 170 participants, [29] is in progress.

### Risks and benefits

The risks for patients taking part in this study are minimal. The plain balloon fistuloplasty is the standard of care and the additional intervention will be the use of a drug-coated balloon or control balloon following this initial dilatation. The drug-coated balloons that will be used are CE marked and there have been no safety concerns with their use.

### Rationale for the current study

Experts in the field are uncertain about the benefit of paclitaxel-coated balloons for AVFs and equipoise remains. In view of the literature discussed above, it is possible that an investigator-led trial of the IN.PACT balloon will show a benefit and hence deliver a different result to the PAVE trial [19] which showed no benefit for the Lutonix balloon. However, this is uncertain, and it is equally possible that it will deliver a negative result. A negative result would also be a worthwhile finding as it is important to establish the presence or absence of efficacy.

As described above under existing research, the case for testing the efficacy of sirolimus-coated balloons in AVFs comprises (a) in vitro data, (b) pre-clinical in vivo data in murine vein graft and AVF models, and (c) two single arm studies showing good outcomes in AVFs.

Although an industry-led trial testing the efficacy of sirolimus-coated balloons in AVFs is in progress, the trial we propose has a larger sample size with more power. Furthermore, the three-armed design in this proposal will provide an answer on the efficacy of both paclitaxel and sirolimus-coated balloons in the same trial. This trial is likely to provide a clear answer regarding the efficacy of drug-coated balloons for AVFs.

### Trial objectives and purpose

The purpose of this trial is to compare the efficacy of additional paclitaxel-coated or sirolimus-coated balloons on outcomes after a plain balloon fistuloplasty to preserve the patency of arteriovenous fistulas used for haemodialysis.

The main objective is to assess the efficacy of paclitaxel-coated or sirolimus-coated balloons in prolonging the time to loss of patency of a treatment segment (segment of vein treated with a fistuloplasty).

Secondary objectives include assessing the efficacy of paclitaxel-coated or sirolimus-coated balloons in prolonging the time to loss of patency at any treatment segment or at any location in the access circuit in a given patient. A further objective is assessing the efficacy of paclitaxel-coated or sirolimus-coated balloons in prolonging the overall survival of the fistula. Other secondary objectives include the number of interventions, fistula-related adverse events and patient quality of life. Secondary objectives that will be assessed with ultrasound are intima-media thickness and the degree of stenosis at 3 months.

## Methods

This protocol has been written using the SPIRIT reporting guidelines [30].

### Study design

The study design is a multicentre randomised controlled trial assessing the superiority of paclitaxel-coated or sirolimus-coated balloons compared to an uncoated control balloon. Following a successful plain balloon fistuloplasty, participants will be randomised to further treatment with a paclitaxel-coated balloon, a sirolimus-coated balloon, or an uncoated control balloon. We will recruit 642 patients, each with one or two treatment segments, over a 3-year period. Patients will remain in the trial and be followed up for 1 year.

Treatment segments will be referred to as treatment segments A or B. Treatment segment A is at the anastomosis or is nearer to the anastomosis than treatment segment B. If there is only one treatment segment, this is always treatment segment A.

### Primary endpoint

Time to loss of treatment segment primary patency (TSPP). The treatment segment is the length of the vein that was in contact with a balloon during the index plain balloon fistuloplasty. TSPP is defined as patency with no re-intervention to the area 5 mm proximal to, within, and 5 mm distal to, the treatment segment. TSPP ends when *any* of the following occurs: (a) clinically driven re-intervention to the treatment segment; (b) thrombotic occlusion considered to be due to restenosis at the treatment segment; (c) surgical intervention that excludes the treatment segment from the access circuit; (d) abandonment of the AVF due to an inability to retreat the treatment segment. In patients with two treatment segments, the primary endpoint will be determined for each treatment segment. The unit of analysis is therefore the treatment segment.

### Secondary endpoints

1. Time to loss of primary patency at any treatment segment. In participants with two treatment segments, this is the time to loss of primary patency at either treatment segment.
2. Time to loss of access circuit primary patency. The access circuit is defined as starting at the arterial anastomosis and ending at the cavo-atrial junction. Access circuit primary patency ends when *any* of the following occurs: (a) access circuit thrombosis, (b) an intervention (either radiological or surgical) anywhere in the access circuit, or (c) the AVF is abandoned due to an inability to treat any lesion.
3. Time to AVF abandonment. AVF abandonment occurs when the AVF is abandoned, regardless of radiological or surgical interventions, with or without a thrombosis event.
4. Total number of radiological or surgical interventions.
5. Adverse events (e.g. thrombosis, infection localised to AVF, rupture of AVF).
6. Patient quality of life as assessed by the mean difference versus the control arm at 6 and 12 months for (a) the EuroQol EQ-5D-5L generic health survey and (b) the vascular access specific VASQoL survey.
7. Mean difference versus control arm at 3 months for (a) intima-media thickness (IMT) and (b) the degree of stenosis measured on ultrasound (only at some sites).

For intima-media thickness (IMT) and the degree of stenosis measured on ultrasound, the unit of analysis is the treatment segment. For the other secondary endpoints, the unit of analysis is the patient.

### Study flowchart

**Fig. 1:**
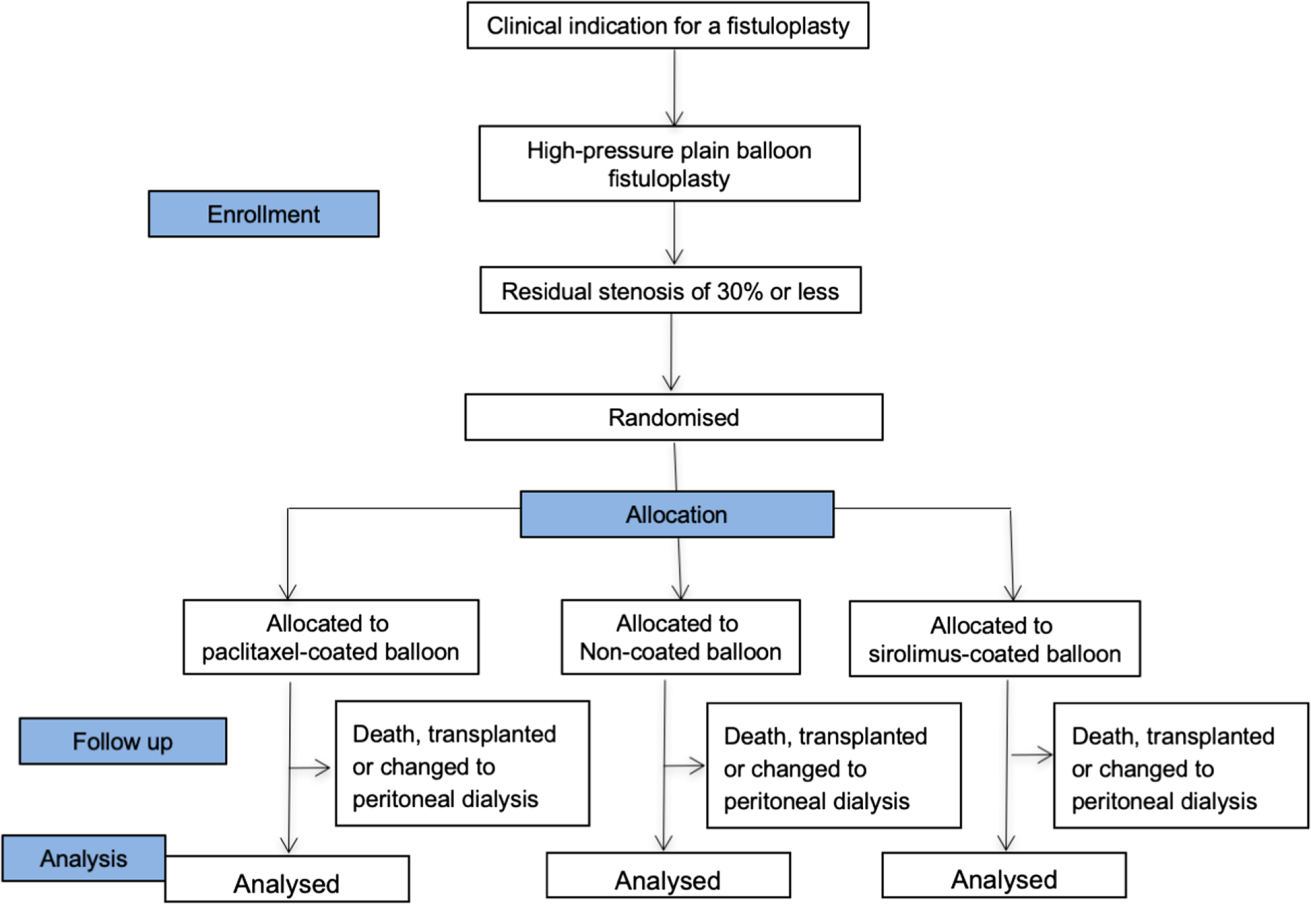
Study flowchart

This is shown in Fig. 1.

### Subject selection

Patients receiving treatment with haemodialysis will be recruited from renal units throughout the UK and at least 20 sites are expected to participate.

### Subject inclusion criteria

1. Patients (18 years or over) who have a surgically formed AVF in the arm which has been used for at least 8 dialysis sessions in the preceding 4 weeks.
2. An indication for a fistuloplasty as determined by the local clinical team.
3. The access circuit is free of synthetic graft material or stents.
4. Patient able to give informed consent.
5. Patient willing and able to comply with all study-related procedures.
6. People who are not breastfeeding, not pregnant, not intending to become pregnant or not intending to father children, within 2 years of study treatment
7. No evidence of active systemic or local (to the fistula) infection.
8. No known hypersensitivity or contraindication to contrast medium which cannot be adequately premedicated.
9. No known hypersensitivity or contraindication to paclitaxel or sirolimus.
10. One or two treatment segments. Each treatment segment will contain one or more stenoses of at least 50%.
11. Each treatment segment will be amenable to treatment with a single drug-coated balloon 8 cm in length or two overlapping drug-coated balloons 4 cm in length.

### Subject exclusion criteria

1. Thrombosed (failed) access circuit at the time of treatment.
2. Location of a stenosis central to the thoracic inlet.
3. The presence of a lesion that has been treated with a plain balloon fistuloplasty where the diameter of the outflow vein is larger than the size of the largest available drug-coated balloon.
4. The presence of a lesion that has been treated with a plain balloon fistuloplasty where the diameter of the outflow vein is considered too small to be treated with the smallest available drug-coated balloon.
5. A significant residual stenosis (more than 30%) at any treated lesion after plain balloon fistuloplasty.
6. Lack of availability of any of the three types of treatment balloon (*Medtronic IN.PACT, Concept Medical MagicTouch* or control) at the required size.

### Subject recruitment

Patients who may be eligible will be identified by surgeons, specialist nurses, radiologists and nephrologists. Informed consent will be obtained by the delegated clinician(s) at each site, following an explanation of the trial procedures and providing the patient with the Participant Information Sheet. The patient will be given sufficient time to read the information, consider the trial and ask questions. Information will be given at the earliest possible opportunity. Less than 24 h notice should be avoided if possible. Consent will be signed before any study-specific procedures are undertaken. If the patient remains potentially eligible for the study before going to radiology for a fistuloplasty, the treating radiologist will be informed. If possible, the person obtaining consent will not be the radiologist who would administer the study treatment if the patient is randomised. If this is not possible then it will not be considered a protocol violation.

### The pre-procedure fistulogram

This will take place immediately prior to the plain balloon fistuloplasty. This will be performed in a dedicated Interventional Radiology suite equipped with digital subtraction angiogram, image overlay/roadmap post-processing capabilities and the ability to capture still and video DICOM file data.

It will be performed through a sheath or cannula placed in the dialysis circuit according to the following specifications: (1) all fistulograms performed as digital subtraction acquisitions at a minimum of 2 frames per second; (2) the entire access circuit from anastomosis to central vein covered in up to 3 stages; (3) medial epicondyle of humerus visible bony landmark on forearm acquisition, acromioclavicular joint on upper arm and central acquisitions; and (4) forearm acquisition to include (i) anteroposterior projection of anastomosis and (ii) oblique projection of anastomosis (specify oblique and craniocaudal angulation).

After the pre-procedure fistulogram, the radiologist will assess all inclusion and exclusion criteria, to decide if the patient remains potentially eligible for the study.

### The plain balloon fistuloplasty procedure

This is performed as standard of care. Prior to treatment, 3000–5000 IU of heparin is administered. For all patients, treatment has two components. The fistuloplasty procedure is performed with a high-pressure balloon having a rated burst pressure of > 18 Atm, unless there is a clinical reason to use a different balloon. The following criteria will be met: (1) sized to nominal vein diameter; (2) ensure obliteration of the lesion waist.

Completion fistulogram I is performed after the plain balloon fistuloplasty to ensure adequate therapy according to the following specifications: (1) All fistulograms performed as digital subtraction acquisitions at a minimum of 2 frames per second; (2) Acquisition that demonstrates the treatment segment(s) matched as close as possible to the respective pre-procedure fistulogram acquisition.

After the plain balloon fistuloplasty and completion fistulogram I, the radiologist will review exclusion criteria 5 (a significant residual stenosis (more than 30%) at any treated lesion after plain balloon fistuloplasty) to assess if the participant remains eligible.

### Randomisation procedures

Randomisation will be at the level of the individual participants, minimising on (i) study site, (ii) whether the AVF has had a previous radiological intervention, (iii) whether the AVF is in the upper arm or forearm and (iv) whether there are one or two treatment segments.

Minimisation will be implemented using an independent web-based randomisation system hosted at the UKCRC registered clinical trials unit at KCL. Site staff will access the service via www.ctu.co.uk using a computer in the angiography room or an office nearby. It will be performed by the radiologist or their nominee, who will log into the system, enter the participant ID number, initials, date of birth, recruiting radiologist, whether the participant has had a previous radiological intervention in the access circuit and whether the AVF is in the upper arm or forearm. Nominees must not be members of the direct care team making decisions about vascular access, and site research team members who randomise will not be involved in the follow-up of that specific patient. Each randomiser will have unique user access, provided by the CTU upon the authorisation of the trial manager, once the delegation of authority form has been completed. Once randomised, the system will automatically generate a confirmation email to the randomiser and trial manager. In patients with two treatment segments, both will be allocated to the same study treatment.

If it is not possible to use the randomisation system, randomisation may occur using the toss of a coin in order to avoid losing the patient from the study. This should only be needed, if at all, in specific and rare situations such as the CTU server being inaccessible. This will be performed by two people and will require at least two coin tosses as follows. heads then heads = control arm; heads then tails = paclitaxel-coated balloon; tails then heads = sirolimus-coated balloon; tails then tails = toss coin again (twice). This process is repeated as needed until the two coin tosses give an outcome other than tails then tails. The CTU must be informed of the coin randomisation as soon as possible.

### Study treatment

In the intervention arm, the second component is the insertion of a single drug-coated balloon (*Medtronic IN.PACT or Concept Medical MagicTouch)* or two overlapping 4 cm drug-coated balloons. If two drug-coated balloons are used, they must overlap by at least 5 mm. Drug-coated balloon(s) must be of identical diameter to or 1 mm bigger than the largest diameter high-pressure plain balloon used. The length of the drug-coated balloon or overlapping drug-coated balloons must be a minimum of 1 cm longer (5 mm at either end) than the entire segment of the vein that has been in contact with a high-pressure plain balloon.

The drug-coated balloon(s) will be inflated to nominal pressure for a minimum of 180 s duration. The duration of inflation will be documented in the eCRF. Instructions for the use of the drug-coated balloon are stringently adhered to ensure appropriate preparation and handling of the device.

In the control arm, an identical procedure is followed, but using a control balloon that is not drug coated. The diameter and length requirements of the control balloon are also identical. Wherever possible, the *Medtronic Admiral Xtreme* balloon will be used. However, if this is not available, a similar control balloon may be used, and this will not be considered a protocol violation.

In both arms, image overlay/roadmap will be utilised to ensure that there is no geographical mismatch between the segments treated with plain balloon or the study treatment balloon. A completion fistulogram is performed (completion fistulogram II) to confirm no angiographically visible effect after treatment with the drug-coated or control balloon, according to the same specifications as completion fistulogram I.

### Measures to avoid bias

A fully blinded trial is not possible due to the differing appearances of the balloons. People who will be aware of the treatment allocation include the treating radiologist and the trial managers (for monthly balloon re-stocking purposes). The patient, other radiologists, the direct care team making decisions related to vascular access, the site research team following up with the patient and the trial statistician undertaking the primary efficacy analysis will remain blinded to treatment allocation. If possible, the person obtaining consent will not be the radiologist who would administer the study treatment if the patient were randomised, as described under subject recruitment. Referral for a repeat procedure will originate from the direct care team who will be unaware of treatment allocation.

A different radiologist to the one performing the index procedure will be involved in subsequent clinical decisions and/or perform a repeat procedure when possible. However, it is not possible to guarantee this. Therefore, the radiologist involved in subsequent decisions and/or performing a repeat procedure may have knowledge of whether the patient was treated with a particular drug-coated or uncoated balloon.

Primary endpoint adjudication: The following applies to any patient while they have at least one treatment segment with primary patency maintained (i.e. a treatment segment that has not yet met the primary endpoint of the trial). (a) For any radiological intervention, the data file(s), in addition to the data file(s) from the index procedure, will be sent to the lead study site with the patient’s name replaced by the trial ID. Files will include the pre-procedure fistulogram, fistuloplasty and all completion fistulograms. The images will be reviewed by a radiologist in the research team who is not based at the same site as the patient. They will decide if they agree with the data on the eCRF (electronic case report form) regarding treatment segment primary patency. Any discrepancies will be discussed with the local radiologist in order to reach agreement. (b) The review process described in (a) will also be followed for any fistulogram performed without a subsequent intervention (see fistulograms performed for a clinical indication). (c) For any surgical intervention or abandonment of the AVF, the eCRF will be reviewed by a member of the research team who is not based at the same site as the patient. They will decide if they agree with the data on the eCRF regarding treatment segment primary patency. Any discrepancies will be discussed with a local investigator in order to reach agreement.

Based on the interim analyses, the DMC and TSC may recommend unblinding the data. The statistician undertaking the primary efficacy analysis will remain blinded.

### Follow up procedures

Study visits will occur every 3 months ± 1 month. These visits will take place either face-to-face or via a telephone conversation. Any face-to-face meetings will usually coincide with dialysis to avoid additional patient travel. At study visits, participants will be asked about any access circuit interventions and changes to peritoneal dialysis, renal transplantation or adverse events. Clinical records will also be reviewed.

EQ-5D-5L and VASQoL will be completed at baseline, 6 months and 12 months. These may be administered in person or over the telephone. If the fistula treated at randomisation is no longer in use, participants will be asked to consider the questions in VASQoL in relation to whatever form of access is in use at the time. Patients may decide they do not wish to be contacted for further study visits, but this does not require withdrawal from the study. They can remain under follow-up with relevant data collected from their medical records. Patients will remain in the trial and be followed up for 1 year.

During follow-up, participants may receive a transplant, change from haemodialysis to peritoneal dialysis, or have their AVF abandoned. They will be considered censored for any AVF patency outcomes that have not been reached at that point but will continue under follow-up in order to collect other data. An exception is a transplant that does not function which results in the patient continuing haemodialysis. In this case, censoring for AVF patency outcomes will not occur.

### Ultrasound assessments

Patients at selected sites will be asked to undergo ultrasound assessments. If they decline, then this will not be considered a protocol violation. Three scans will be performed for each treatment segment. These will be (i) 30 days or less, prior to the study intervention, (ii) immediately post intervention and (iii) at 3 months (± 1 month). A high-resolution linear array transducer will be used. B-mode ultrasound images will be acquired at the points of stenosis or at the site of the previous balloon angioplasty, when restenosis has not been demonstrated and will be used to quantify IMT. Measurements will include the outer-to-outer wall vessel diameter and luminal diameter. Volume flow will be measured in the brachial artery and peak systolic velocity will be measured at points of stenosis within each treatment segment. A standard operating procedure will be available and will be followed.

### Fistulograms performed for a clinical indication

In patients with at least one treatment segment that has not yet reached the primary endpoint of the trial, the fistulogram (pre-procedure if there is a subsequent intervention) will follow the same specifications as the pre-procedure fistulogram. Data file(s) will be sent to the lead site for review as specified for primary endpoint adjudication (see measures to avoid bias), even if an intervention is not performed.

### End of study definition

End of study is defined as the last participant’s last follow-up. The trial may be prematurely discontinued by the Sponsor, Funder, Chief Investigator or TSC based on new safety information or for other reasons given by the DMC, TSC, and REC. The trial may also be prematurely discontinued due to lack of recruitment or upon advice from the TSC who will advise on whether to continue or discontinue the study and make a recommendation to the sponsor. If the trial is prematurely discontinued, active participants will be informed, and no further participant data will be collected.

### Blood tests

No blood tests are required as part of the trial. The most recent pre-procedure full blood count and CRP results will be entered on the eCRF.

### Assessment of safety

The PAVE-2 protocol does not fall within the Clinical Trial Regulations and therefore is not a drug trial. In addition, the drug-coated balloons are CE-marked medical devices, so prior regulatory approval from the MHRA is not needed. Safety reporting will be in keeping with the requirements for research other than Clinical Trials of Investigational Medicinal Products.

A Serious Adverse Event (SAE) is an untoward occurrence that (a) results in death, (b) is life-threatening, (c) requires non-elective hospitalisation or prolongation of existing hospitalisation, (d) results in persistent or significant disability or incapacity, (e) consists of a congenital anomaly or birth defect or (f) is otherwise considered medically significant by the investigator.

For this trial, SAEs will only be reported if they are related to the study treatment or the access circuit that has been treated and if they are unexpected. Examples of events that will not be considered SAEs include (but are not limited to) the following: Respiratory infections, fluid overload, diverticulitis, gastrointestinal infection, bowel obstruction, cholecystitis, falls, syncope, myocardial infarction, fractures, foot infections, skin infection (unrelated to the access circuit), urinary tract infection,

We do not expect any SAEs to be related to the study treatment or the access circuit that has been treated. Therefore, any SAEs that are considered to be related to the study treatment or the access circuit that has been treated will be reported. They will be reported by the local investigators on the SAE form to the Chief Investigator, as soon as they become aware and within 24 h at most*.* Although it is not an SAE, any pregnancy or fathering of children that occurs during follow-up will be reported via the SAE system.

Since the study treatment is local and not systemic, non-serious adverse events will be defined as events that local principal investigator (PI) considers are directly related to the study treatment or the access circuit that has been treated. These should be recorded throughout the trial and will be captured in the eCRF at each study assessment. Deaths of study participants will be recorded. All collected SAEs and AEs will be reported in the primary trial publication.

### Ethics reporting

Reports of SAEs will be reviewed by the CI within 24 h to see if the local PI considers that the event is related to the research procedure and unexpected (a SUSAR) and if so, it will be onward reported to the REC and DMC within 15 days.

### Data monitoring committee

The membership will be decided by the CI and approved by the NIHR. The DMC includes a statistician and two other independent experts. They will receive a report of recruitment, serious and non-serious adverse events and a summary of accumulated clinical data from the trial statistician, and will meet in person, online, or by telephone. They will report to the TSC and will meet at least annually during the study. Additional meetings may take place at the time of interim analysis or in case of recruitment issues. The DMC is advisory to the TSC. The DMC charter will be drafted and agreed prior to recruitment. A Trial Statistician will prepare reports for the DMC.

### Trial steering committee

The TSC membership will be decided by the CI and approved by the NIHR. The chair will be an independent expert. Members will include the CI and a patient representative. At least 75% of members will be independent. The TSC will meet at least annually during the study. Additional meetings may take place at the time of interim analysis or in case of recruitment issues. The TSC is an executive committee. Terms of reference of the TSC will be agreed upon and documented prior to the start of recruitment. The Trial Manager will prepare reports to the TSC.

### Ethics and regulatory approvals

This protocol and related documents were reviewed by the London-Hampstead Research Ethics Committee (REC). HRA and Health and Care Research Wales (HCRW) approval was given (23/LO/0625).

### Subject compliance

Subject compliance is not expected to be an issue as the study treatment is administered at one time after randomisation.

### Subject withdrawal

Participants have the right to withdraw from the study at any time for any reason. It is understood by all concerned that an excessive rate of withdrawals can render the study uninterpretable; therefore, unnecessary withdrawal of patients should be avoided. Should a patient decide to withdraw from the study, all efforts will be made to report the reason for withdrawal as thoroughly as possible. Patients may remain in the trial with no further contact from the research team (see above under follow up procedures). Therefore, we anticipate that withdrawal should be very uncommon.

### Protocol compliance

Any instances where the allocated treatment is not administered will be promptly reported and investigated to establish the reason and minimise future occurrences. Following randomisation, there are no restrictions on usual care.

### Data to be collected

Data to be collected at each visit is indicated in the schedule of treatments (Table 1). Sources will include the clinical notes and discussion with the patient. At baseline, the following definitions will be used in the medical history; coronary artery disease is defined as previous myocardial infarction, coronary artery bypass surgery, percutaneous coronary artery intervention or significant disease on a coronary angiogram. Peripheral vascular disease is defined as previous surgery (bypass/amputation), radiological intervention, or evidence of disease on angiography/ultrasound. A stroke will be assumed to have been ischaemic if it is uncertain whether it was ischaemic or haemorrhagic.

**Table 1:** Schedule of treatments for each visit

Activity	Baseline	Procedure						Month 3	Month 6	Month 9	Month 12
Patient registration and consent	x										
Medical history (including indication for fistuloplasty)	x										
Consideration of eligibility	x										
Confirmation of potential eligibility with radiologist	x										
Pre-procedure fistulogram		x									
Plain balloon fistuloplasty			x								
Completion fistulogram I				x							
Randomisation					x						
Study treatment						x					
Completion fistulogram II							x				
Ultrasound (at selected sites only)	x						x	x			
Follow up assessments								x	x	x	x
Quality of life assessments (EQ-5D-5L and VASQoL)	x								x		x

### Data handling and record keeping

During the study, only the direct care team or site research team will have access to participants’ identifiable data. Any paper documents with personal data will be held in a locked filing cabinet in a locked office and retained for a minimum of 5 years following the end of the study.

Pseudonymised clinical and research data for the study will be stored on the eCRF system, hosted at the King’s Clinical Trials Unit, KCL for at least 15 years. The eCRF (InferMed MACRO) is GCP and FDA 21 CFR Part 11 compliant. Data entry staff at site will be provided with unique usernames and passwords to the system and will be trained in data entry by the trial manager. The trial manager will visit sites to review data on the system, raise discrepancies and confirm source data verification checks. All requests for access to the data entry system must be authorised by the trial manager. All requests for data exports must be authorised by the trial statistician. The trial manager will work with the CI and the trial statistician to ensure data is checked and cleaned on an ongoing basis and will confirm all data checks have been completed before database lock.

The investigators and the institutions will permit trial-related monitoring, audits, REC review, and regulatory inspections (where appropriate) by providing direct access to source data and other relevant documents (i.e. patients’ case sheets, blood test reports, and X-ray reports). Record keeping will be the responsibility of the investigators.

## Statistical considerations

### Sample size calculation

The 12-month target lesion primary patency from the three large trials [17–19] was 44, 46.3 and 58.8% respectively in the control groups, giving a mean of 50% and we have assumed this for the control group. Given the lack of certainty based on the results of previous trials, we have selected a higher hazard ratio of 0.6, which equates to a 12-month cumulative target lesion primary patency of 66% in both treatment groups, which allows us to detect smaller differences than observed in previous trials. Based on an alpha of 0.05 and a power of 90%, we would require 199 per group to detect a difference of 16% in the cumulative target lesion primary patency at 12 months between one of the treatment groups and the control group using the exponential test of survivor functions [31]. We will not have the power required to compare the two treatment groups directly. With three arms to the trial, we would therefore need 597 patients. Patients will be censored if they receive a kidney transplant, switch to peritoneal dialysis, die or withdraw their consent. Based on our experience in the PAVE trial, we estimate this at 10% by 12 months. Conservatively allowing for 15% censoring due to loss to follow-up we aim to randomise 642 patients according to the Lachin and Foulkes method [31]. Given the group sequential procedure used in the analysis, whereby formal interim analyses consider the primary outcome when 33% and 66% of expected total follow-up data are available, this is the maximum sample size, and the actual sample size will be smaller where the decision is to stop one or more arms early.

We have based the estimated sample size on individual patients, but the inclusion and analysis by treatment segments (accounting for shared frailty) rather than at the patient level will mean that the power exceeds that stated above. Two treatment segments in the same patient will not be independent but we do not have sufficient data to estimate the intraclass correlation for shared frailty. Therefore, we cannot directly estimate the extent to which this will increase the power of the study. However, we explored the impact of this based on locally collected data indicating a mean of 1.4 treatment segments per patient, which indicated that there would be a 10–20% increase in efficiency for intraclass correlations ranging between a rho of 0.3 and 0.5.

Statistical analyses will be conducted in accordance with the intention to treat principle (including all randomised patients according to their allocated treatment group) by a statistician masked to group allocation, following a pre-specified analysis plan. All analyses will use a 5% significance level.

### Statistical analysis

Cox-proportional hazard models will be used to estimate hazard ratios for the time to loss of TSPP (primary outcome) for each treatment group relative to the control group. We will not have the power required to compare the two treatment groups directly. Estimates will be presented both as hazard ratios and absolute differences in the cumulative loss of treatment segment primary patency at 12 months. Kaplan–Meier survivor functions will be used to graphically describe the rate of loss of patency. The period of observation will start on the day of randomisation and end on the day when loss of TSPP is recorded, censored at 12 months, or on the last date that status was known if that is earlier. The treatment group will be included as dummy coded variables with covariates included reflecting the minimisation factors included in the randomisation procedure. Analysis will be based on treatment segments rather than participants and a shared frailty (i.e. random effect) will be estimated to account for the clustering of treatment segments within patients.

Secondary outcomes regarding time to loss of patency at any treatment segment, time to loss of access circuit primary patency and time to AVF abandonment will be analysed using the same approach as the primary outcome, except that analysis will be based on participants.

Patient-reported outcomes (EQ-5D-5L and VASQoL [32]), assessed at baseline, 6 and 12 months, will be analysed using linear mixed effects models. Mean differences between each treatment group and the control group at each time point will be estimated by including dummy coded variables relating to the treatment group, time of assessment, and group-by-time interaction terms in the model. Additional covariates will include minimisation factors used in the randomisation procedure and the baseline level of the outcome variable. A random intercept will be estimated to account for the repeated observations within individuals.

In the biological plausibility analysis, where the outcome is neointimal hyperplasia at 3 months, linear mixed effects models will also be used. Analysis will be based on treatment segments rather than participants. These will follow a similar approach as described above, except one observation per treatment segment is recorded at a single time point so there will be no time-related variables and the random intercept will not be included. If TSPP has been lost before 3 months, ultrasound data will not be available. However, we predict that, where a group has a greater loss of treatment segment primary patency at 3 months, the lesions still patent will have an increase in neointimal hyperplasia. This would therefore still support biological plausibility.

### Pilot and interim analysis

An internal pilot will consider recruitment rates at 9-months. We anticipate that at 18 months of recruitment, 50% of patients will have been recruited with around 33% of the expected total person-time follow-up available (and thus the total number of events) and at 30 months 66% of total person-years follow-up available. When 33% of the expected total follow-up data is available, an interim analysis will be performed to test superiority for each treatment compared to the control group with a number of possible outcomes: stop two arms (and thus the trial), continue all three arms, and stop one arm. Decisions will be made separately for each treatment based on recommendations from the DMC with the control arm continuing throughout. When testing superiority an alpha spending function following the DeMets-Lan approach will be implemented based on the information fraction available at the time of the analysis to control the overall alpha level at 5%. When 66% of the total person-years follow-up is available, a second interim analysis will be performed with the same considerations and decisions.

### Ethical considerations

This study was formally peer-reviewed during the funding application. The trial will be conducted in compliance with the principles of the Declaration of Helsinki (1996), the principles of GCP and in accordance with all applicable regulatory requirements including but not limited to the UK Policy Framework for Health and Social Care Research. Informed consent is described in above.

The Chief Investigator will submit a final report at the conclusion of the trial to the sponsor and the REC. Annual progress reports will be submitted to the main REC for the study. Patients were involved in the planning of this study and were applicants on the funding application. Independent TSC members include a patient representative.

### Reporting and dissemination

It is intended that the results of the study will be reported and disseminated at international conferences and in peer-reviewed scientific journals. The chief investigator will be the senior author on trial publications and the collaborators named in the protocol will be co-authors. Investigators from all contributing sites will be named in the primary trial publication.

## Trial status

Trial recruitment started in May 2024 with the first participant randomised on 17 June 2024. The approximate date for completion of recruitment is January 2027. This publication is based on protocol version 2.3, dated 3 June 2024.

## Data Availability

Following publication of the main trial outcome, the trial dataset will be shared following any reasonable request and subject to a data sharing agreement in accordance with the consent provided by participants.

## Abbreviations

AVF: Arteriovenous fistulas
AVG: Arteriovenous grafts
CI: Chief Investigator
eCRF: Electronic Case Report Form
CVC: Central venous catheter
DMC: Data Monitoring Committee
ISRCTN: International Standard Randomised Controlled Trial Number
JLA PSP: James Lind Alliance Priority Setting Partnership
MRSA: Methicillin-resistant *Staphylococcus aureus*
MSSA: Methicillin-sensitive *Staphylococcus aureus*
PAVE: Paclitaxel-assisted balloon Angioplasty of Venous stenosis in hEmodialysis access
PI: Principal investigator
Participant: An individual who takes part in a clinical trial
RCT: Randomised controlled trial
REC: Research Ethics Committee
SAE: Serious adverse event
SONG-HD: Standardised Outcomes in Nephrology–Haemodialysis
TLPP: Target lesion primary patency
TSPP: Treatment segment primary patency
TSC: Trial Steering Committee
